# Trends in *Neisseria gonorrhoeae* Antimicrobial Resistance over a Ten-Year Surveillance Period, Johannesburg, South Africa, 2008–2017

**DOI:** 10.3390/antibiotics7030058

**Published:** 2018-07-12

**Authors:** Ranmini Kularatne, Venessa Maseko, Lindy Gumede, Tendesayi Kufa

**Affiliations:** 1 Centre for HIV & Sexually Transmitted Infections, National Institute for Communicable Diseases, Johannesburg 2131, South Africa; venessam@nicd.ac.za (V.M.); lindyg@nicd.ac.za (L.G.); tendesayikc@nicd.ac.za (T.K.); 2 Department of Clinical Microbiology & Infectious Diseases, Faculty of Health Sciences, University of the Witwatersrand, Johannesburg 2195, South Africa; 3 School of Public Health, Faculty of Health Sciences, University of the Witwatersrand, Johannesburg 2195, South Africa

**Keywords:** *Neisseria gonorrhoeae*, antimicrobial resistance

## Abstract

Background: In South Africa, sexually transmitted infections (STIs) are managed through a syndromic approach at primary healthcare centres (PHCs). *Neisseria gonorrhoeae* is the predominant cause of male urethritis syndrome. We describe antimicrobial resistance patterns and trends in *Neisseria gonorrhoeae* during a ten-year surveillance period at a large PHC in Johannesburg. Methods: *Neisseria gonorrhoeae* was cultured from genital discharge swab specimens obtained from consenting adult patients presenting at the Alexandra Health Centre in Johannesburg between 2008 and 2017. Isolates were tested for antimicrobial susceptibility by Etest™ (cefixime, ceftriaxone, ciprofloxacin) or agar dilution (penicillin, tetracycline, azithromycin). Results: During the period of surveillance, high-level resistance prevalence increased from 30% to 51% for penicillin (*p*-value for trend < 0.001), 75% to 83% for tetracycline (*p*-value for trend = 0.008), and 25% to 69% for ciprofloxacin (*p*-value for trend < 0.001). Analysis did not reveal high-level resistance to spectinomycin or a minimum inhibitory concentration (MIC) creep for extended-spectrum cephalosporins, and the prevalence of intermediate-resistance to azithromycin was less than 5%. Conclusions: High prevalence resistance to penicillin, tetracycline, and ciprofloxacin in *N. gonorrhoeae* obviates their use in future national treatment algorithms for genital discharge. It is essential to continue monitoring for emerging resistance to currently recommended antimicrobial therapy in this rapidly evolving pathogen.

## 1. Introduction

Gonorrhoea is a major public health concern worldwide. The infection has a short incubation period of a few days, as well as a high transmission efficiency, and leads to a fivefold increase in HIV transmission and complications such as pelvic inflammatory disease and infertility, which compound the global health burden [1]. The World Health Organisation (WHO) 2012 prevalence data for curable sexually transmitted infections revealed that the estimated global prevalence of gonorrhoea among women aged 15–49 years was 0.8% (95% uncertainty interval 0.6–1.0%) and among males aged 15–49 years old the prevalence was 0.6% (0.4–0.9%) [2]. These estimates corresponded to an incidence of 78 million (53–110 million) new cases of gonorrhoea.

*Neisseria gonorrhoeae*, which is an obligate human pathogen, has displayed an alarming propensity to acquire resistance through genetic mechanisms (both chromosomal and plasmid-mediated) to all sequential first-line antimicrobial agents used over the years [3]. Penicillin was first used in the 1940s and tetracycline from the 1950s to the 1980s, but high-level plasmid-mediated resistance to both agents was being described by the 1980s. Quinolones were introduced in the early 1980s, but resistance emerged in the Asia-Pacific region and spread globally [4]. Antimicrobial resistance does not appear to confer a fitness cost as resistant strains continue to predominate globally following withdrawal of the antimicrobial from clinical use [4]. Extended-spectrum cephalosporins (ESCs) were regarded as the last antimicrobial class suitable for widespread single-dose single-agent treatment. Cefixime is the most potent oral ESC and was also considered effective for the treatment of pharyngeal gonorrhoea [4,5]. In Japan in the 1990s, use of a variety of oral ESCs with suboptimal efficacies and inadequate dosing regimens led to ultimate treatment failure with cefixime [6]. By 2010, clinically confirmed cefixime treatment failures had been described in Europe and North America [7]. In 2007, reports of ceftriaxone treatment failure emerged from Australia, and by 2010 were being described in Japan and Western Europe [7]. Importantly, all were cases of pharyngeal gonorrhoea. Spectinomycin, an aminocyclitol, was used in the 1980s as first-line therapy for the treatment of gonorrhoea. However, clinical treatment failures and in vitro resistance were being reported within a few years of use [4]. Azithromycin is usually recommended only in dual combination therapy for gonorrhoea, as azithromycin monotherapy may result in high-level resistance to the antimicrobial via sequential point mutations in 23S rRNA [8].

In South Africa, sexually transmitted infections (STIs) are managed through a syndromic approach at primary healthcare centres (PHCs), which ensures that treatment is given for the major causative pathogens based on clinical manifestations. Data showing the distribution of STI syndromes among males and females attending PHCs in South Africa reveal that male urethritis syndrome (MUS) and vaginal discharge syndrome (VDS) comprise the bulk of STI presentations [9]. Syndromic management of STIs has resulted in a loss of specimen-taking skills among healthcare workers as well as a lack of development of optimised and standardised laboratory protocols for microbial culture and antimicrobial resistance testing. It has also led to a lack of ongoing investment in laboratory infrastructure in many regions. Periodic aetiological surveillance of STI syndromes is essential to update and validate the existing syndromic management guidelines. The Centre for HIV and STIs (CHIVSTI) at the National Institute for Communicable Diseases (NICD) in Johannesburg has co-ordinated microbiological surveillance in patients presenting to sentinel PHCs since 2006. Results indicate that *Neisseria gonorrhoea* is the predominant cause of MUS (70–85%) and is present in 10–20% of symptomatic VDS cases [10]. 

The WHO Global Gonococcal Antimicrobial Surveillance Program (GASP) was relaunched in 2009 to timely monitor the trends of antimicrobial resistance in *Neisseria gonorrhoeae* and improve knowledge on potential resistance mechanisms through laboratory testing [11]. South Africa is a participating country, and the STI laboratory at CHIVSTI has conducted gonococcal resistance surveys annually in Johannesburg since 2008. In May 2015, following endorsement by the 68th World Health Assembly for a Global Action Plan that aims to enhance global antimicrobial resistance surveillance activities, the WHO developed the Global Antimicrobial Resistance Surveillance System (GLASS) and listed *Neisseria gonorrhoeae* as a priority pathogen.

We describe *Neisseria gonorrhoeae* antimicrobial resistance patterns and trends from Johannesburg STI sentinel surveillance over a ten-year period spanning 2008–2017.

## 2. Results

### 2.1. Characteristics of Participants

In total, *Neisseria gonorrhoeae* isolates from 2445 unique individuals were tested between 2008 and 2017. Demographic and clinical characteristics of participants whose genital discharge could be attributed to *Neisseria gonorrhoeae* are given in Table 1. Most participants were male (92.2%), black African (99.7%), and of self-reported heterosexual orientation (99.8%). Median age of participants was 27 years. Over 30% of females and 25% of males gave a history of STI syndrome in the preceding year. Almost one-third of males and over 50% of females were HIV-infected, and this difference was statistically significant (*p* < 0.001).

### 2.2. Neisseria gonorrhoeae Antimicrobial Resistance Profiles by Calendar Year

*Neisseria gonorrhoeae* E-test minimum inhibitory concentrations (MICs) for cefixime and ceftriaxone are available for every consecutive year from 2008 to 2017 (Table 2). Fewer isolates were tested using agar dilution, and data are available for 2008, and subsequently from 2011 to 2017 (Table 2). Due to increasing trends of high-level resistance in *N. gonorrhoeae* noted for ciprofloxacin, penicillin, and tetracycline, antimicrobial susceptibility testing (AST) for these antimicrobials was discontinued in 2017.

**Figure 1:**
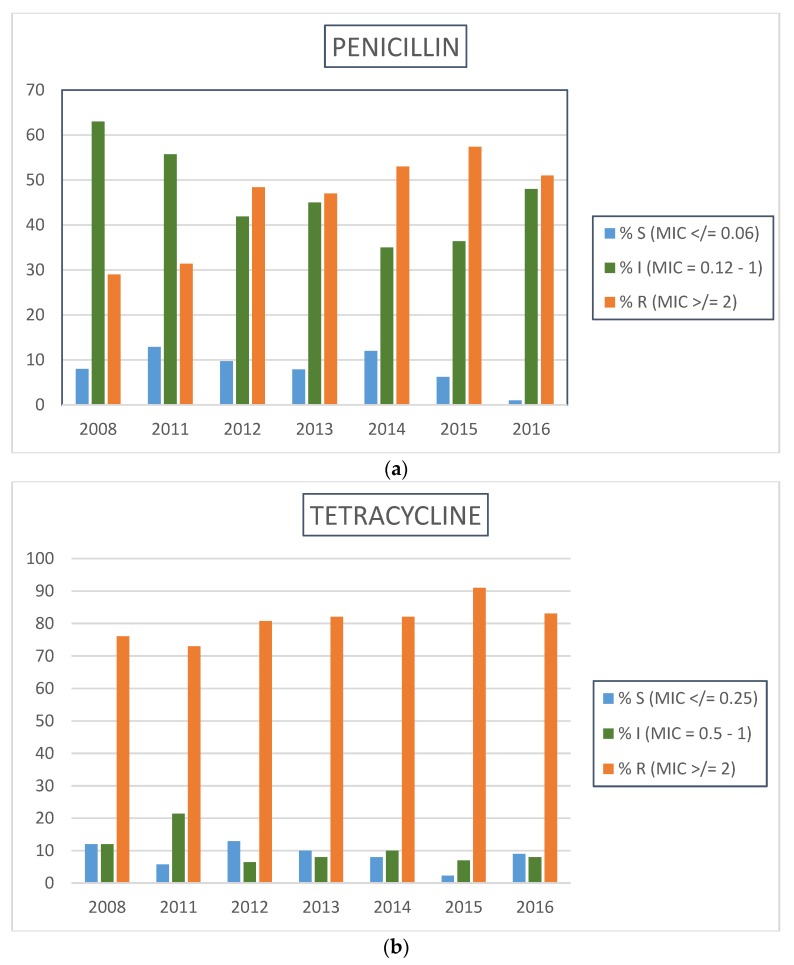

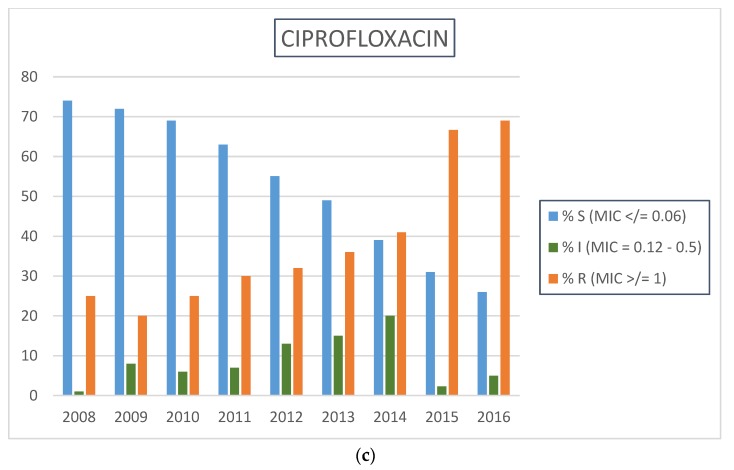
Prevalence of resistance by calendar year for (**a**) penicillin, (**b**) tetracycline, and (**c**) ciprofloxacin in *Neisseria gonorrhoeae*, Johannesburg, 2008–2016. Minimum inhibitory concentration (MIC) breakpoint criteria (µg/mL) as per Clinical Laboratory Standards Institute (CLSI) 2018; 28th Edition.

During the period under review, there was a general increase in the prevalence of *Neisseria gonorrhoeae* resistance to penicillin, tetracycline, and ciprofloxacin (Figure 1). Between 2008 and 2016, the prevalence of high-level resistance rose from 30% to 51% for penicillin (x^2^ *p*-value = 0.009; *p*-value for trend < 0.001) and 75% to 83% for tetracycline (x^2^ *p*-value = 0.013; *p*-value for trend = 0.008). Between 2008 and 2016, the prevalence of high-level resistance to ciprofloxacin rose exponentially from 25% to 69% (x^2^ *p*-value < 0.001; *p*-value for trend < 0.001).

The Clinical and Laboratory and Standards Institute (CLSI) defines decreased susceptibility to extended-spectrum cephalosporins (DS ESC) as an MIC ≥ 0.5 µg/mL [12], whereas the European Committee on Antimicrobial Susceptibility Testing (EUCAST) uses a resistance breakpoint that is one double-dilution lower at ≥ 0.25 µg/mL [13]. More than 99% of isolates were sensitive to ESCs (Table 3). Decreased susceptibility to cefixime was not observed using interpretive CLSI criteria, whereas it was seen in one isolate from 2013 (0.4%) using EUCAST breakpoints. Two isolates from 2009 exhibited resistance to ceftriaxone using EUCAST criteria (0.6%); one of these (0.3%) also showed reduced ceftriaxone susceptibility according to CLSI breakpoints (Table 4). These two isolates were not available for further molecular analysis. 

Although trend analysis shows that there is a significant difference in MIC_50_ between years, our data do not reveal an MIC creep for cefixime or ceftriaxone, i.e., a sustained and progressive increase by year in MIC_50_ and MIC_90_.

The Clinical and Laboratory Standards Institute has not established interpretive criteria for azithromycin. EUCAST defines intermediate-level azithromycin resistance as an MIC of 0.5 µg/mL, and full resistance as MIC > 0.5 µg/mL, based on an epidemiological cut-off of 1 µg/mL for wild-type *Neisseria gonorrhoeae* isolates [13]. The US Gonococcal Isolate Surveillance Project (GISP) uses a breakpoint of ≥2 µg/mL to define elevated MICs to azithromycin, and increased likelihood of treatment failure [7].

Intermediately-resistant azithromycin MICs were observed across all years, except in 2016 (Table 5). Full resistance according to EUCAST criteria was observed in isolates only from 2008; the highest MIC observed in that year was 1 µg/mL. Intermediate resistance to azithromycin decreased from 9.4% (22/233) in 2008 to 2.5% (3/122) in 2017; and an MIC creep was not apparent.

During the ten-year period of surveillance, the majority of gonococcal isolates (>95%) displayed full susceptibility to spectinomcyin (Table 6). From 2012 onwards, all isolates were uniformly susceptible and an MIC creep for spectinomycin has not been observed.

## 3. Discussion

This surveillance report describes resistance trends to various antimicrobials used in past and current gonorrhoea treatment regimens over a ten-year surveillance period at a large primary healthcare centre in Johannesburg. Participants were mostly young adults, with the majority self-reporting heterosexual orientation, and belonged to a high risk-population for STI—a significant proportion had a history of recent STI and were HIV-infected.

Our data reveal that penicillin and tetracycline are unlikely to be included in any future genital discharge treatment algorithms in South Africa. In South African isolates, high-level penicillin resistance in *Neisseria gonorrhoeae* was found to be plasmid-mediated: a novel beta-lactamase producing the “Johannesburg” plasmid was identified and these penicillinase-producing isolates were discovered to be clonally related [14]. Similarly, both American- and Dutch-type tetracycline-resistant *Neisseria gonorrhoeae* (TRNG) plasmids have been detected in South African isolates, and confer high-level resistance to the drug [15].

Escalating *N. gonorrhoeae* ciprofloxacin resistance was seen in Johannesburg and Cape Town when data from 2004 and 2007 were compared [16]. In Johannesburg, there was a 1.9-fold increase in resistance prevalence from 11% to 32%; and in Cape Town, a 2.9-fold increase from 7% to 27%. The World Health Organization (WHO) recommends a change of empirical treatment for gonorrhoea when the resistance threshold reaches 5% [17]. In 2008, the South African syndromic management guidelines for genital discharge were formally revised to incorporate oral cefixime as first-line therapy for gonorrhoea [18]. A sustained increase in high-prevalence resistance to ciprofloxacin was observed during our surveillance period, despite withdrawal of the antibiotic as a treatment option for genital discharge in 2008. This likely represents a fitness benefit conferred by type II topoisomerase mutations associated with ciprofloxacin resistance [4], as well as antimicrobial selection pressure from continued use of ciprofloxacin at PHCs for other indications, such as acute cystitis. 

The primary resistance determinant for extended-spectrum cephalosporins (ESCs) is a specific alteration in the *penA* gene encoding penicillin binding protein 2 (PBP2). This is thought to occur through acquisition and recombination into its genome of foreign gene sequences from commensal *Neisseria* species residing in the oropharynx [19]. This transformation gives rise to a mosaic *penA* gene encoding a mosaic PBP2 with reduced target affinity for ESCs: the MICs of cefixime are increased proportionately more than those of ceftriaxone [20].

In South Africa, in 2012, the first two cases of cefixime resistance associated with cefixime treatment failure were described in two patients presenting with persistent urethral discharge [21]. Genetic characterization of the two isolates using *Neisseria gonorrhoeae* multi-antigen sequence typing (NG-MAST) and multi-locus sequence typing (MLST), revealed identical sequence types (NG-MAST ST4822 and MLST ST1901) which represent a multi-drug resistant clone associated with cefixime treatment failure and global spread. Both patients were in the men-who-have-sex-with-men (MSM) risk group. There are two factors that could lead to the spread of resistance in this key population: high risk sexual behaviour and participation in international sexual networks; and the presence of pharyngeal gonorrhoea, which is typically asymptomatic. Gonococci residing in the pharynx are at a survival advantage due to differential concentrations of antimicrobials at this site, and the opportunity for genetic exchange with oropharyngeal commensal *Neisseria* species. An additional two cases of resistance to cefixime were identified in MSM residing in Cape Town and East London, respectively (D. Lewis, unpublished data).

In 2009, the world’s first confirmed extensively-drug resistant (XDR) *Neisseria gonorrhoeae* infection was reported in Japan [22]. The gonococcal strain, isolated from the pharynx of a commercial sex worker (CSW), displayed high-level resistance to both cefixime (MIC = 8 µg/mL) and ceftriaxone (MIC = 2–4 µg/mL). It was also resistant to several other classes of antimicrobials, including fluoroquinolones, macrolides, and tetracycline.

Following these reports, in accordance with WHO recommendations, there was a national change in recommended first-line therapy for gonorrhoea from oral cefixime to injectable ceftriaxone (250 mg single-dose) in 2014. Additional dual therapy with oral azithromycin (1 g stat) was recommended [23]. This was a pro-active and pre-emptive approach, endorsed by the WHO, to limit the emergence of XDR *Neisseria gonorrhoeae* particularly in areas where there is a general lack of surveillance in key populations, such as MSM and CSW [24]. 

High-level resistance to spectinomycin is relatively rare, and has been attributed to a single nucleotide polymorphism in 16S rRNA as well as mutated ribosomal protein S5 [25]. Although spectinomycin appears to be a viable alternative in our setting, it is not listed on the South African Essential Medicines List for primary healthcare centres; and has reduced efficacy against pharyngeal gonorrhoea [4]. We have identified low prevalence intermediate-resistance to azithromycin (MIC 0.5 µg/mL) among gonococcal isolates in recent years. Although clinical effectiveness of azithromycin for urethral and endocervical infections has been estimated to be >95% [26], it is recommended only in dual therapy due to the ease of resistance development to macrolide monotherapy. Resistance has been described even with use of higher dose (2 g) azithromycin monotherapy [27], and this dose is associated with increased gastro-intestinal side-effects. Successful and sustained spread of a high-level azithromycin-resistant (MIC ≥ 256 µg/mL) clone of *Neisseria gonorrhoeae* (NG MAST ST 9768) has been described in England [28]. The outbreak, which began among young heterosexuals in Leeds in November 2014, had by 2016 become geographically dispersed to the North and South of the country and spread to MSM sexual networks. Whole genome sequencing revealed the presence of mutations in all four alleles of the 23S rRNA gene in the majority of isolates, and raised concerns that high-level resistance may develop stepwise from low-level resistance characterised by the presence of mutation in a single allele, especially in the setting of azithromycin selection pressure.

Failure of dual ceftriaxone–azithromycin therapy has been described from the United Kingdom, with persistence of asymptomatic pharyngeal gonorrhoea in a heterosexual man treated for urogenital symptoms in 2014 [29]. The patient was infected with an XDR strain, which had acquired multiple resistance mechanisms to both ceftriaxone and azithromycin. Molecular epidemiology identified the isolate as MLST ST1901 and a new NG-MAST ST 12133, which belongs to a genogroup that is associated with extensive drug-resistance and is spreading in Japan. More recently, Public Health England issued a report of a heterosexual man with a test-of-cure pharyngeal gonococcal isolate showing resistance to ceftriaxone (MIC 0.5 µg/mL) and high-level resistance to azithromycin (MIC > 256 µg/mL), with possible links to a female sexual contact in South East Asia [30].

The ease with which *Neisseria gonorrhoeae* develops drug resistance means that antimicrobial stewardship strategies are urgently needed: rational, standardized, and regulated prescription practice for genital discharge syndrome. Research and development initiatives should include funding for novel antimicrobials with unique mechanisms of action, facilitating their incorporation into appropriate therapeutic regimens. 

Limitations of this surveillance study include the lack of in-depth information on behavioural risk characteristics of participants, as well as being conducted in largely heterosexual populations at a single clinical site. There is a need for accurate rapid diagnostics for gonorrhoea that would facilitate screening for asymptomatic and extra-genital infection in high-risk population groups such as MSM. Additionally, allocation of resources is required for enhanced local surveillance strategies in order to better understand transmission dynamics and inform control efforts. These would include the design and implementation of activities to increase detection of treatment failure cases and extragenital (pharyngeal) infections at primary and secondary healthcare level, as well as sustained antimicrobial surveys (including test-of-cure) in key populations.

## 4. Methods

### 4.1. Patient Recruitment

*Neisseria gonorrhoeae* antimicrobial resistance surveys were conducted at the Alexandra Health Centre (AHC) in Johannesburg each year between 2008 and 2017. The AHC is a community-based PHC in Johannesburg, the largest city in South Africa and the provincial capital of the most populous province of the country (Gauteng Province). It is located in a Johannesburg sub-district that has a population of over 600,000. The centre offers a variety of community oriented primary healthcare services, including comprehensive STI syndromic management, HIV counselling and testing, and prevention services such as condom promotion and distribution. From 2008 to 2014, national microbiological surveillance activities required the systematic recruitment of consecutive adult males and females 18 years or older presenting with a new episode of genital discharge. From 2015 onwards, the NICD GERMS-SA surveillance protocol was implemented, and involved sampling of approximately 150–200 males presenting with urethritis for the isolation of at least 100 viable gonococcal isolates per year from the sentinel site. Written informed consent was followed by a short nurse-administered questionnaire, which collected demographical and behavioural information of participants. Each participant was given a unique survey number, which was delinked from personal identifiers. 

### 4.2. Sample Collection

Swab specimens of visible urethral and vaginal discharge were collected by the surveillance nurse from consenting adult males and females, respectively. The urethral and endocervical specimens were inoculated directly on New York City agar (Diagnostic Media Products, National Health Laboratory Service), and placed in a holding candle jar prior to same-day transfer to the STI Reference laboratory at the NICD.

### 4.3. Laboratory Procedures

At the reference laboratory, the inoculated agar plates were incubated at 37 °C in CO_2_ and examined daily for growth up to a total of three days. Colonies suspicious for *Neisseria gonorrhoeae* were definitively identified using biochemical tests (oxidase and catalase) as well as a specific slide co-agglutination immunological assay—the Phadebact^®^ Monoclonal GC test (MKL Diagnostics AB, Kung Hans Vag, Sollentuna, Sweden). *Neisseria gonorrhoeae* isolates were tested for susceptibility to antimicrobials by E-test™ (bioMérieux, Marcy-l’Étoile, France) for cefixime, ceftriaxone, and ciprofloxacin; or agar dilution for penicillin, tetracycline, azithromycin, and spectinomycin according to established standard operating protocols. Minimum inhibitory concentrations were interpreted according to criteria recommended by the Clinical Laboratory Standards Institute (CLSI) [12], or for azithromycin susceptibility, according to European Committee on Antimicrobial Susceptibility Testing (EUCAST) clinical breakpoints [13]. For purposes of quality control a panel of WHO reference strains of *N. gonorrhoeae* with known MICs for each antimicrobial was included in every batch of clinical isolates tested.

### 4.4. Data Management and Statistical Analysis

Data from clinical questionnaires and results of gonococcal antimicrobial susceptibility testing were entered into a survey-specific Access© (Microsoft, Seattle Washington) database. Data were exported into STATA 14^®^ (Stata Corporation, College Texas, Texas, TX, USA) for analysis. The enrolled participants were described using frequencies and proportions for categorical data, and medians and interquartile ranges (IQRs) for continuous variables. Analysis of susceptibility trends in *Neisseria gonorrhoeae* involved determination of the relative prevalence of susceptibility and resistance for those antimicrobials to which resistance is well-established. Chi-square tests were used to determine whether the difference in high-level resistance prevalence between calendar years reached statistical significance. For antimicrobials that are currently recommended for use, such as extended-spectrum cephalosporins and azithromycin, MIC_50_ (the minimum concentration needed to inhibit 50% of isolates), MIC_90_ (the minimum concentration needed to inhibit 90% of isolates), and maximum MICs were determined by year. The K-sample test was used to test for equality of medians (MIC_50_) across calendar years, while piecewise logistic regression with splines at each calendar year was used to test for trends in high-level resistance across calendar years.

## 5. Conclusions

Antimicrobial susceptibility profiles and trends for *Neisseria gonorrhoeae* in Johannesburg over a period of ten years reveal that high-prevalence resistance to penicillin, tetracycline, and ciprofloxacin obviates the use of these agents in national empiric therapy guidelines for syndromic management. The prevalence of resistance to ceftriaxone is <1%, and intermediate resistance to azithromycin <5%, validating continued use of both in dual therapy for gonorrhoea. Enhanced national culture-based surveillance is essential to detect emerging resistance, particularly in key populations.

## Figures and Tables

**Table 1:** Demographic and clinical characteristics of participants with gonorrhoea, Johannesburg, 2008–2017.

Characteristic	Males (2254)	Females (191)	All (2445)
Age in years (median, IQR) *	28 (24–32)	24 (23–30)	27 (24–32)
Ethnic group (black African) ^β^ (%)	1921 (99.7)	164 (100)	2085 (99.7)
History of STI syndrome in past 12 months (%)	580 (25.8)	63 (31.3)	643 (26.3)
Heterosexual orientation ^α^ (%)	1907 (99.8)	161 (99.4)	2068 (99.8)
HIV positivity ^µ^ (%)	655 (32.5)	104 (52.0)	759 (34.3)
Year of enrolment (%)			
2008	309 (13.7)	29 (15.2)	338 (13.8)
2009	305 (13.5)	19 (10.0)	324 (13.2)
2010	287 (12.7)	29 (15.2)	316 (12.9)
2011	260 (11.5)	38 (19.9)	298 (12.2)
2012	273 (12.1)	21 (11.0)	294 (12.0)
2013	221 (9.8)	28 (14.7)	249 (10.2)
2014	208 (9.2)	27 (14.1)	235 (9.6)
2015	137 (6.1)	0 (0.0)	137 (5.6)
2016	128 (5.6)	0 (0.0)	128 (5.7)
2017	128 (5.7)	0 (0.0)	128 (5.7)

* Available for 2418 (2229 males and 189 females), ^β^ available for 2091 participants (1929 males and 162 females), ^α^ available for 2073 participants (1913 males and 160 females), ^µ^ available for 2213 participants (2023 males and 190 females). STI: sexually transmitted infection; IQR *: interquartile range.

**Table 2:** Number of *Neisseria gonorrhoeae* isolates tested for susceptibility to various antimicrobials by calendar year, Johannesburg, 2008–2017.

	Antimicrobials and AST Method	
Year	Cefixime (CXM), ceftriaxone (CTR), ciprofloxacin (CIP) E-test MIC	Azithromycin (AZM), penicillin (PEN), tetracycline (TET), spectinomycin (SPC) Agar dilution MIC
2008	338 (CTR and CIP only)	233
2009	324	0
2010	316	0
2011	298	70
2012	294	31
2013	249	77
2014	235	93
2015	136	125
2016	128	113 (CIP included)
2017	128 (CXM and CTR only)	122 (AZM and SPC only)

MIC = minimum inhibitory concentration; AST: antimicrobial susceptibility testing.

**Table 3:** Cefixime MIC trend analysis, Johannesburg, 2008–2017.

Year	No. of Isolates	MIC_50_	MIC_90_	Maximum MIC	% with MIC = 0.125	*% with MIC = 0.25	% with MIC >/= 0.5
2009	324	<0.016	0.016	0.064	0	0	0
2010	316	<0.016	<0.016	0.016	0	0	0
2011	297	<0.016	<0.016	0.016	0	0	0
2012	294	<0.016	<0.016	0.016	0	0	0
2013	249	0.016	0.016	0.25	0	0.4 (*n* = 1)	0
2014	235	<0.016	0.016	0.047	0	0	0
2015	137	0.016	0.032	0.064	0	0	0
2016	128	<0.016	0.016	0.016	0	0	
2017	128	<0.016	0.016	0.125	0.8 (*n* = 1)	0	0

MIC = minimum inhibitory concentration (µg/mL). Resistance defined as MIC ≥ 0.25 μg/mL (European Committee on Antimicrobial Susceptibility Testing (EUCAST) 2018; version 8.0). *p*-value for equality of medians across years < 0.001; *p*-value for trend < 0.001.

**Table 4:** Ceftriaxone MIC trend analysis, Johannesburg, 2008–2017.

Year	No. of Isolates	MIC_50_	MIC_90_	Maximum MIC	% with MIC = 0.125	% with MIC = 0.25	% with MIC >/= 0.5
2008	338	0.002	0.004	0.008	0	0	0
2009	324	0.003	0.006	0.38	0	0.3 (*n* = 1)	0.3 (*n* = 1)
2010	316	0.002	0.006	0.032	0	0	0
2011	298	0.003	0.004	0.012	0	0	0
2012	294	0.003	0.004	0.012	0	0	0
2013	228	0.003	0.006	0.064	0	0	0
2014	235	0.004	0.008	0.047	0	0	0
2015	136	0.003	0.006	0.012	0	0	0
2016	135	0.004	0.008	0.032	0	0	0
2017	128	0.003	0.008	0.032	0	0	0

MIC = minimum inhibitory concentration (µg/mL). Resistance defined as MIC ≥ 0.25 μg/mL (EUCAST 2018; version 8.0). *p*-value for equality of medians across years < 0.001; *p*-value for trend = 0.001.

**Table 5:** Azithromycin MIC trend analysis, Johannesburg, 2008–2017.

Year	No. of Isolates	MIC_50_	MIC_90_	Maximum MIC	% Susceptible MIC </= 0.25	% Intermediately-Resistant MIC = 0.5	% Resistant MIC > 0.5
2008	233	0.128	0.5	1	86.3	9.4	4.3
2011	70	0.128	0.25	0.5	94.3	5.7	0
2012	31	0.128	0.25	0.5	93.6	6.4	0
2013	77	0.25	0.25	0.5	90.9	9.1	0
2014	93	0.064	0.25	0.5	97.9	2.1	0
2015	125	0.128	0.25	0.5	97.6	2.4	0
2016	113	0.128	0.25	0.25	100	0	0
2017	122	0.064	0.25	0.5	97.5	2.5	0

MIC breakpoint criteria (µg/mL) as per EUCAST 2018; version 8.0. *p*-value for equality of medians across years < 0.001; *p*-value for trend = 0.007; x^2^ for trend in AZM sensitivity < 0.001.

**Table 6:** Spectinomycin MIC trend analysis, Johannesburg, 2008–2017.

Year	Number Tested	MIC_50_	MIC_90_	Maximum MIC	% Susceptible (MIC < 32)	% Intermediately-Resistant (MIC = 64)	% Resistant (MIC ≥ 128)
2008	233	16	32	64	99.6	0.4	0
2011	70	16	32	64	98.6	1.4	0
2012	31	16	32	32	100	0	0
2013	77	16	32	32	100	0	0
2014	93	16	16	32	100	0	0
2015	125	32	32	32	100	0	0
2016	113	16	16	32	100	0	0
2017	122	16	16	32	100	0	0

MIC breakpoint criteria (µg/mL) as per CLSI 2018; 28th Edition. *p*-value for equality of medians across years < 0.001; *p*-value for trend < 0.001.
